# Buffalo and the A. M. A. Meeting

**Published:** 1911-07

**Authors:** 


### Buffalo and the A. M. A. Meeting

THE Medical Society of the County of Erie at its June meeting decided not to make any effort to secure for Buffalo the 1912 meeting of the American Medical Association. Under the circumstances the convention bureau of the Chamber of Commerce will not take any action. The chance of securing this meeting was bright up to the point where it became necessary to determine what the city would do toward defraying the necessary expenses. The Medical Society of the County of Erie very properly could not undertake the burden and in the absence of anything definite in the way of financial assistance from the city or the Chamber of Commerce it was decided to suspend activities.

The Chamber of Commerce, by the way, had taken no step toward bringing the meeting of the association to Buffalo other than to acquiesce in the plan. There were no city or chamber representatives at the Los Angeles meeting to urge Buffalo as the next meeting place. Other cities wanting the meeting had representatives on the ground; other chambers of commerce were represented and brought the advantages of their locations prominently forward. Buffalo which has been loudly and frequently bespeaking for itself the meeting, and telling in round-about fashion of its beauties and its advantages, did nothing but sit down at the last minute and forget it.

One of the Buffalo newspapers stated the reason no effort would be made to secure the meeting was "because it was deemed improbable that the $10,000 fund estimated for expenses could be raised among the local doctors," which statement is absurd. The physicians of Buffalo would have not the slightest difficulty in raising the amount stated. It would be a matter of comparatively few days to raise the money.

The profession is entirely within its rights in declining to assume so great a convention responsibility without assistance from the city or the chamber of commerce of material character. And, judging by recent performances of the board of councilmen in dealing with a forthcoming convention of a national beneficial organisation in holding up proposed city appropriation for the sanitation of the meeting place, it would appear to a casual observer that the medical profession was wise in its determination to not plunge headlong into any convention places without assurances of official assistance in preparing for the great meeting which the American Medical Association of 1912 would have been in Buffalo.

